# Effects of Levetiracetam Treatment on Hematological and Immune Systems in Children: A Single-Center Experience

**DOI:** 10.3390/children12080988

**Published:** 2025-07-28

**Authors:** Yasemin Özkale, Pınar Kiper Mısırlıoğlu, İlknur Kozanoğlu, İlknur Erol

**Affiliations:** 1 Department of Pediatrics, Adana Dr. Turgut Noyan Teaching and Medical Research Center, Baskent University Faculty of Medicine, 06790 Adana, Turkey; 2 Acıbadem Labmed Clinical Laboratories, 34752 İstanbul, Turkey; 3 Department of Pediatric Neurology, Adana Dr. Turgut Noyan Teaching and Medical Research Center, Baskent University Faculty of Medicine, 06790 Adana, Turkey

**Keywords:** levetiracetam, complete blood count, immunoglobulin levels, lymphocyte subsets

## Abstract

**Objective:** The interactions between the central nervous system (CNS) and the immune system suggest that immune mechanisms may be effective in the pathogenesis of epilepsy and epileptic seizures. Although studies on the natural immune response and epilepsy are continuing, it is not yet clear whether the interaction of the current immune system is due to epilepsy itself or antiepileptic drugs (AEDs), since epileptic patients also use AEDs There are a limited number of studies that have reported an increased incidence of upper respiratory tract infections (URTIs) in patients during levetiracetam (LEV) treatment. Therefore, we aimed to report our experience regarding the effect of LEV monotherapy on the complete blood count (CBC), immunoglobulin (Ig) levels, and lymphocyte subgroups in the interictal period in children and adolescents with epilepsy. **Methods:** This study enrolled 31 children who presented with epilepsy and underwent LEV monotherapy for at least one year (patient group) and 43 healthy children (control group). The CBC parameters (hemoglobin (hb), lymphocytes, leukocytes, neutrophils, and platelets), Ig levels (IgA, IgM, IgG, and IgE), and lymphocyte subsets (CD3, CD4, CD8, CD4/CD8 ratio, CD19, CD56, NKT cells, and Treg cells) were measured and compared between the two groups. The patients were also investigated regarding the frequency and types of infections that they experienced in the first month and first year of the study, and these data were compared between the patient group and the control group. In addition, the same parameters and the frequency of infection were compared among the patient subgroups (focal and generalized seizures). **Results:** The results of the present study indicate that there were no significant differences in the CBC parameters, lymphocyte subsets, or Ig levels between the patient group and the control group. The comparison among the patient subgroups was similar; however, the CD4/CD8 ratio was lower in the patient subgroup with focal seizures. In addition, there were no significant differences in the frequency or type of infections experienced one month and one year of the study between the patient group and the control group, and likewise for the patient subgroups (focal and generalized seizures). **Conclusions:** The present study demonstrated that LEV monotherapy did not increase the incidence of infection, and there were no significant effects on the CBC or on the humoral or cellular immune system in epileptic children. These findings also suggest that the CD4/CD8 ratio among lymphocyte subgroups is lower in patients with focal seizures. However, the epilepsy subgroups had a relatively small sample size; therefore, further prospective studies involving a larger patient population are needed to establish the association between LEV monotherapy and lymphocyte subgroups in patients with epilepsy.

## 1. Introduction

It is the task of the acquired immune system to create defenses against infectious substances, and this defense is carried out with lymphocytes and antibody responses. A defense mechanism develops at the humoral and/or cellular level for each agent. Humoral immunity is composed of antibodies produced by B lymphocytes, and cellular immunity is composed of T lymphocytes [1]. It has not been clarified whether the changes in the humoral immunity following an epileptic seizure are due to an exogenous cause, such as an infection, or to an interaction between the CNS and the immune system. The interactions between the CNS and the immune system suggest that immune mechanisms may be effective in the pathogenesis of epilepsy and epileptic seizures [2]. Although studies on the natural immune response and epilepsy are continuing, it is not yet clear whether the interaction of the current immune system is due to epilepsy itself or AEDs, since epileptic patients also use AEDs. In a limited number of studies, it was observed that the humoral immunity was influenced in children who had epileptic seizures [2]. Although the effect of AEDs on the immune system continues, it is thought that valproic acid (VPA), carbamazepine (KBZ), phenytoin, vigabatrin, diazepam, and LEV impact immune system activity by altering the synthesis and release of some molecules and cytokines, thus affecting humoral and cellular immunity [3]. However, the results of studies on changes in the humoral and cellular immunity due to AEDs such as phenytoin, KBZ, and VPA are contradictory [4,5,6].

Levetiracetam is used as either an adjunctive therapy or a monotherapy for epilepsy in children [7]. However, there is a growing body of clinical evidence suggesting that LEV treatment leads to an increased risk of infections, especially upper URTIs, including pharyngitis, rhinitis, coughs, and sinusitis. Furthermore, LEV treatment may result in an increased risk of various hematological side effects [7,8,9,10,11]. While an increased risk of URTIs has been associated with lymphopenia in some studies, this relationship has not been observed in other studies. LEV has been associated with negative effects on the antiviral functions of the immune system and an increase in socialization after seizure control [8,12,13,14,15]. Therefore, we aimed to report our experience about the frequency and types of infections experienced by patients who were treated with LEV monotherapy, and the effect of LEV on hematological factors and the immune system in patients with epilepsy and subgroups.

## 2. Materials and Methods

This study included 31 patients from 4 to 16 years of age who were diagnosed with epilepsy and received LEV monotherapy for at least one year at the Başkent University Adana Dr. Turgut Noyan Research and Application Center Pediatric Neurology Department, and 43 healthy children in the same age group. The exclusion criteria were as follows: a history of chronic disease (renal insufficiency, liver dysfunction, immunodeficiency, autoimmune disease, hematological disease, etc.), drug use (as this could have an effect on the immunological system), an infection during the 2 weeks leading up to the study, developmental retardation, malnutrition, who had seizures in the last month leading up to the study, regular blood transfusions, and the receival of treatments with an immunomodulatory effect on the immune system, such as an interferon, intravenous immunoglobulin, or steroids, within six months of the start of the study.

Infections were classified as URTIs, lower respiratory tract infections, acute gastroenteritis, or urinary tract infections. The frequency of seizures in the last year, the seizure type (according to the ILAE classification), the duration of LEV treatment, the initial dose, and the current dose were recorded.

The CBC parameters were grouped as normal, high, or low according to the age groups reported in Table 1 [16].

For the flow cytometric analysis, two separate tubes were prepared. The first tube contained monoclonal antibodies targeting lymphocyte subpopulations, while the second tube included monoclonal antibodies specific to regulatory T cells. All the monoclonal antibodies were obtained from Becton Dickinson (BD). The flow cytometric analysis was performed using an eight-color, three-laser FACS Canto II cytometer (BD Biosciences, San Jose, CA, USA), and the data were analyzed with the FACS DIVA software (BD Biosciences). The percentages and absolute counts (per microliter) of lymphocyte subsets and regulatory T cells were determined through a flow cytometric evaluation.

### Lymphocyte Subgroup Analysis

Regulatory T Cell (Treg) Analysis: For the analysis of regulatory T cells, lymphocytes were gated using the CD45 vs. SS plot. T lymphocytes were selected from the CD3 vs. SS plot, followed by the selection of CD4+ T helper cells from the CD4 vs. SS plot. Treg cells were identified by analyzing the expression of CD25 and FoxP3 within the CD4+ T cell population. As with the previous analysis, the percentage of Treg cells was recorded, and the absolute Treg cell counts per microliter were calculated using the patients’ total leukocyte counts.

Interpretation Based on Pediatric Reference Values: It is important to note that the lymphocyte subgroups analyzed using flow cytometry do not have universally established reference values in pediatric populations; instead, the average values vary with age and developmental stage. Therefore, the lymphocyte subset results were interpreted within age-appropriate reference ranges and categorized as normal, elevated, or decreased accordingly [17].

**Figure 1:**
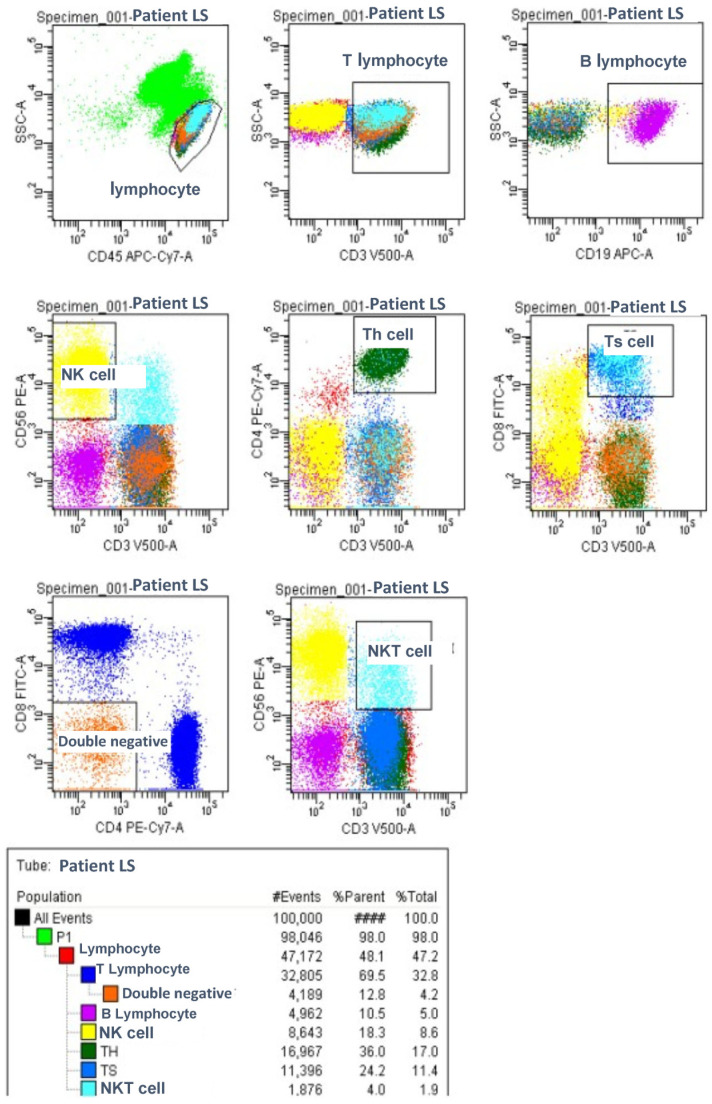
Lymphocyte Subgroups Analysis.

**Figure 2:**
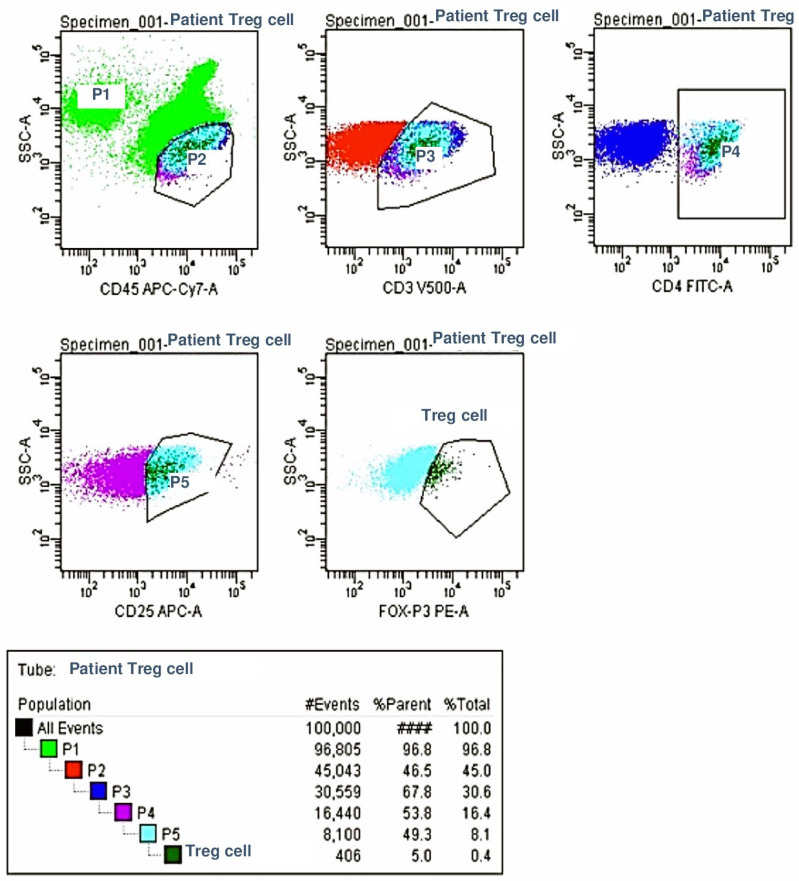
Regulator Cell Analysis.

A graphical study of the lymphocyte subgroups and Treg cells is shown in Figure 1 and Figure 2.

Since the IgA, G, and E levels vary according to age, and the IgM level varies according to gender, the values were assessed according to these distinctions and evaluated as normal, high, or low [18].

Ethical approval was obtained from the Başkent University Clinical Research Board, under decision number KA14/295.

## 3. Statistical Analyses

The SPSS 17.0 package program was used for the statistical analyses. The Win-Epi 2.0 program was used for sample calculations. Categorical measurements were summarized as numbers and percentages, and continuous measurements are represented by the mean and standard deviation (or median and minimum–maximum where necessary). A chi-square test or Fisher’s test statistic was used to compare the categorical variables. In the comparison of continuous measurements between the groups, the independent variables were subjected to a *T*-test and the variables that did not meet the parametric distribution prerequisite were subjected to a Mann–Whitney U test. A repeated measures analysis was used for repeated measurement comparisons. Spearman’s correlation coefficient or Pearson’s correlation was assessed. The main purpose of this study was to compare the patient and control groups. In order to test the homogeneity of the data in some parameters, the difference between the seizure types was examined, but there were no statistical differences.

## 4. Results

Based on the above criteria, there were no significant differences between the patient and control groups with respect to age or sex (Table 2). Of the 31 epilepsy patients, 16 were diagnosed with genetic epilepsy, 9 with structural/metabolic epilepsy, and 6 had epilepsy of an unknown type. Generalized seizures were observed in 75.2% of the patients, while focal seizures were observed in 25.8%.

Table 3 summarizes the frequency and types of infections in the first month and first year of the study between the two groups; no statistically significant differences were found (*p* = 0.39 and *p* = 0.82, respectively). The same result was also observed among the epilepsy subgroups (focal and generalized seizures) (*p* = 0.56 and *p* = 0.39, respectively).

Table 4 summarizes the relationships among the duration of LEV use, the initial dose, and the frequency of infections in the year leading up to the study. The mean duration of LEV treatment was 2 years (1–4 years). VPA treatment was started as the first drug in eight patients, but the treatment was switched to LEV monotherapy after the seizures were controlled. There were no significant relationships among the duration of treatment, the initial dose of LEV, and the frequency of infections in the year prior to the study.

Table 5 summarizes the results for the CBC parameters in the patient and control groups. There were no significant differences between the two groups (*p* > 0.05) or between the focal and generalized seizure subgroups in the CBC parameters (hb (*p* = 0.29), leukocytes (*p* = -), lymphocytes (*p* = 0.29), neutrophils (*p* = 0.69), and platelets (*p* = 0.53)).

Within the epilepsy group, the IgA, IgM, IgG, and IgE levels in the subgroups with epilepsy of a genetic, structural/metabolic, or unknown cause were not statistically different, and the same result was observed for the focal and generalized seizure subgroups (Table 6).

The CD_3_^+^, CD_8_^+^, CD_4_^+^, CD_56_^+^, CD_19_^+^, NKT cell levels, and CD_4_/CD_8_ ratio were compared between the patient and control groups; no differences were found, as presented in Table 7 and Table 8. When the focal and generalized seizure subgroups were compared, the same results were found except for the CD_4_/CD_8_ ratio, which was significantly lower in the focal seizure group (*p* = 0.006).

Table 9 summarizes the results for the CD4CD25foxP3 percentage and the number of cells per microliter for the patient and control groups. There were no significant differences between the two groups (*p* = 0.78, *p* = 0.70).

## 5. Discussion

Our study shows that there were no significant differences in the frequency or types of infections between patients who were treated with LEV monotherapy and a control group in the month and year leading up to the study. Additionally, no significant differences were found between LEV monotherapy and the control group in terms of the CBC, Ig levels, or lymphocyte subgroups, including T cells. Within the epileptic subgroups, no significant differences were found between the focal and generalized seizure subgroups for the CBC, Ig levels, or lymphocyte subgroups, but the CD_4_/CD_8_ ratio was lower in patients within the focal seizure subgroup.

Several clinical studies have shown an increase in infections such as URIs, including pharyngitis and rhinitis, in patients receiving LEV treatment [8,9,10,19,20]. Piña-Garza et al. reviewed 152 epileptic patients who were treated with LEV for 48 weeks, aged from 1 month to 4 years, and found that a fever (39.5%) was the most common adverse event, followed by URIs (27.6%), vomiting (18.4%), and nasopharyngitis (17.1%) [21]. In another study, the author investigated 103 patients with partial-onset seizures who received LEV monotherapy for 48 weeks between the ages of 4 and 16 years and found that URIs were the third most common adverse effect, with a prevalence of 21.4% [22]. Similar findings have also been reported in adult patients in various clinical studies, with up to 13.4% compared to 7.5% in the placebo group [23]. However, in the present study, we found no statistical differences in the frequency of infections in patients using LEV monotherapy and no correlation between the duration of drug use, the starting dose, or the frequency of infections between the patient and control groups. Since our study had a relatively small sample size and the mechanism of increased URIs in these patients is not fully understood, the association between URIs and LEV monotherapy needs to be further investigated with a larger series.

Indeed, several studies have suggested an association between lymphopenia and a high incidence of URIs in patients receiving LEV monotherapy [8,12]. For example, Dinopoulos et al. followed 22 patients aged 2–15 years receiving LEV monotherapy and found a significant decrease in lymphocyte counts in the 6th month of treatment, while Attilakos et al. revealed lymphopenia in the 12th month of LEV monotherapy [7,24]. On the other hand, some studies have stated that there was no change in the lymphocyte count in patients who received LEV monotherapy in the 6th or 12th month of treatment, as in our study [8,24,25,26]. In the present study, no significant differences were found between the serum hb, leukocyte, lymphocyte, neutrophil, or platelet levels in patients who had undergone LEV monotherapy for at least one year. However, Attilakos et al. reported long-term hematological side effects of LEV monotherapy and showed that the lymphocyte and platelet counts were significantly decreased and the neutrophil counts were significantly increased after 12 months of LEV monotherapy [24]. In our previous study, LEV monotherapy was shown to cause a decrease in the white blood cell counts in the first month, which returned to normal in the sixth month of treatment [27]. Bachmann et al. found that 52 patients receiving LEV monotherapy had a statistically significant decrease in their platelet counts compared to the control group in a study of 251 adult patients. Although the mechanism was not clear, they speculated that it could be associated with the SV2A protein [12]. However, Sahaya et al. speculated that thrombocytopenia could be immune-mediated; this hypothesis was supported by Kimland et al., who observed irregular antibodies for platelets in the blood of patients who were treated with LEV [28,29]. On the other hand, French et al. reported that the Hb and Hct values were lower in patients receiving LEV monotherapy compared to a control group, but Attilakos et al. showed a significant increase in the Htc and MCV values in the 12th month after LEV monotherapy [15,24]. Since the methods, statistical analyses, and results of these studies are different from each other, and the studies generally used small numbers of patients, further studies are needed to elucidate whether or how CBC parameters are affected in patients using LEV monotherapy.

The relationship between AEDs and humoral immunity has also been evaluated in a number of studies. Some studies have suggested that patients who have received VPA, CBZ, phenytoin, phenobarbital, or lamotrigine have changes to their Ig levels and IgG subgroups [30,31,32]. Svalheim et al. reported that there was a significant decrease in the IgG subgroup levels in patients who received lamotrigine and CBZ, but there were no changes in patients who received LEV monotherapy between epileptic and healthy adults [30]. On the other hand, Kalantari et al. determined that the Ig levels were decreased in patients with LEV monotherapy, and the authors speculated that a reduction in Igs could cause an increased susceptibility to infections in these patients [31]. Interestingly, Azar et al. reported a 19-year-old patient with a brain abscess who received LEV treatment, but the authors found a significant decrease in the Ig levels, while the B and T lymphocytes and NK cells were normal. The authors replaced LEV monotherapy with topiramate and observed that the Ig levels gradually increased to normal. The authors speculated that hypogammaglobulinemia may occur in children receiving LEV treatment, and that this could be a risk factor for an increased number of infections [33]. Therefore, they suggested that the serum Ig levels should be examined before and after LEV treatment. In contrast, we found no significant differences in the Ig levels between the patients who received LEV monotherapy and the control group. There was a slight decrease in the IgA levels of each patient between the patient and the control groups, and it was observed that these patients did not differ from the other patients in terms of their infection frequency. The factors that may have contributed to these contradictory results include the small patient numbers, the different ages of the patients included in the study, and differences in the laboratory methods used by different centers.

Studies on the effects of AEDs on cellular immunity are ongoing, and the results are conflicting [34,35]. VPA and LEV may have an impact on seizure-induced immunological changes. Bauer et al. showed that the NKT cell levels were increased and the CD_4_^+^ T lymphocyte levels were decreased in patients with temporal lobe epilepsy after a seizure, and while the decrease in the CD_4_^+^ T lymphocyte levels was more prominent in patients receiving VPA treatment, the NKT cell increase was lower in patients receiving LEV. The authors reported that the changes in all of the patients returned to normal values 24 h after the seizure. As a result, they showed that VPA treatment has an increasing effect and LEV treatment has a decreasing effect on the changes in cellular immunity caused by seizures [35]. Since our study was conducted on patients who had not experienced a seizure in the last month, the effect of seizures on the immune system was excluded. Another study investigated the proinflammatory cytokines and lymphocyte subgroups in the interictal period, as in our study [34]. An increase in the percentage of B lymphocytes in patients using lamotrigine and an increase in CD_4_^+^ T lymphocytes in patients using VPA were detected. The authors reported a decrease in the CD_8_^+^ T lymphocyte level in patients using LEV, but the reduction was not statistically significant [34]. The results should be interpreted with caution, as the number of patients was limited, the duration and dose of medication were not evaluated, and the patients were taking additional AEDs, which are known to have an effect on the immune system. The authors reported that it was not possible to decide whether the effects of AEDs on the systemic immune system are pathophysiological or epiphenomenal. Our study was performed on epileptic children and adolescents using LEV monotherapy, and the fact that the age, gender, duration of drug use, and dose were taken into consideration in the statistical evaluation increases the reliability of the results.

Guenther et al. examined the CD_4_^+^, CD_8_^+^, and CD_4_^+^ CD_25_^+^ lymphocyte levels in patients over 15 years of age with active epilepsy, 15 of whom used VPA (12 as a monotherapy) and 21 of whom used LEV (17 as a monotherapy) in the pre-treatment period and in the third month of treatment. In the patients using VPA, there was a significant decrease in the CD_4_^+^ lymphocyte percentage in the third month of treatment, while no significant changes were detected in the cytokine levels. In the patients using LEV, the authors found a significant decrease in the CD_4_^+^ CD_25_^+^ cell percentage without any changes in the cytokine levels [23]. The effects of LEV on the CD_8_^+^ lymphocyte functions and apoptosis are unknown. Li et al. investigated the effect of LEV and VPA treatments on proliferation, apoptosis, CD107a/b mobilization, and perforin release in 15 healthy adults using in vitro methods by adding different doses of LEV (5 and 50 mg/L) and VPA (10 or 100 mg/L) to blood samples. The authors showed that VPA therapy has no effect on the cytotoxic functions of CD_8_^+^ lymphocytes, but high doses of VPA reduce spontaneous apoptosis. In contrast, they found that high doses of LEV reduced the perforin release of CD_8_^+^ lymphocytes and that the use of LEV had no effect on the apoptotic effect and proliferation of CD_8_^+^ lymphocytes [13]. In our study, the cytotoxic functions of CD_8_^+^ lymphocytes were not examined, and it was observed that the patients who used LEV had normal CBCs, Ig levels, and lymphocyte subgroup levels. Since the frequency and type of infections in our study group did not differ from the control group, it may be thought that the cytotoxic functions of CD_8_^+^ lymphocytes were also unaffected. However, the average LEV dose was 22 mg/kg/day in our study. Further studies on this subject are needed, along with a search for the effect of higher doses of LEV on the cytotoxic functions of CD_8_^+^ lymphocytes.

Chen et al. reported that the B and T lymphocyte subgroups were effective at hippocampal neurodegeneration in mice with kainic acid-induced seizures. In their study, CD_8_^+^ lymphocytes were shown to be effective against the neurocytotoxic function. They also reported that CD_4_^+^ and B lymphocytes have protective effects against kainic acid-induced excitotoxic damage [36]. Immunological phenomena have been described by some other studies in patients with focal epilepsy [34,37]. Bauer et al. studied lymphocyte subgroups, serum epinephrine levels, and leukocyte, neutrophil, and NK cell levels in 22 patients with temporal lobe epilepsy who were using various AEDs. They showed that leukocytes, neutrophils, lymphocytes, NK cells, and the epinephrine levels increased, and the CD_4_^+^ T lymphocyte levels decreased immediately after a seizure. These changes were more prominent in patients with hippocampal sclerosis. They also noted that the post-seizure changes were significant only in patients with complex partial seizures. At a 24-h follow-up, the changes were shown to return to normal values. The authors concluded that postictal immunological changes may be related to epinephrine release, and that the greater immune response in patients with mesial temporal lobe epilepsy may be related to the close relationship between the mesial temporal tracts and the sympathetic nervous system [35]. However, in our study, acute changes that may have occurred as a result of seizures were excluded by ensuring that there was at least one month between the time of recruitment and the time of the seizure. Despite the small number of patients, we found that the CD_4_/CD_8_ ratio of the patients who had focal seizures was significantly lower than that of the generalized seizure group in our study. These results support the hypothesis that impaired T cell functions may be effective in focal epilepsy without the effect of acute seizures. Indeed, Eeg Olofsson et al. also showed that the CD4/CD8 ratio and NK activity decreased in the interictal period of epileptic patients [37]. The mechanisms, such as localized neuroinflammation, CD4+ T cell exhaustion, hippocampal sclerosis, or region-specific immune dysregulation, may explain the role of the immune system in focal epilepsy [35,36]. On the other hand, Nowak et al. did not detect any differences between lymphocyte subgroups and the cytokine levels among patients with focal or generalized seizures [34]. Further large-scale studies are warranted to explain T lymphocyte function in patients with focal seizures.

Treg cells are responsible for maintaining peripheral tolerance, ensuring immune balance, preventing autoimmune diseases, and limiting chronic inflammatory diseases. Treg cells also have the ability to inhibit the functions of CD_4_^+^ CD_25_^+^ T cells, dendritic cells, NK and NKT cells, and B lymphocytes. FoxP3 is a transcription factor that has a critical role in determining the development and function of CD_4_^+^ CD_25_^+^ T cells [38]. There are limited studies in the literature examining Treg cells in epilepsy patients. Li C et al. showed that Treg cell numbers in the peripheral blood are significantly lower in epileptic children compared to a control group, and it was reported that these abnormal values in the peripheral blood may be effective in the pathogenesis of epilepsy [13]. However, no studies in the English literature have investigated the relationship between drug use and Treg cells in epileptic patients. The present study showed that there were no statistically significant differences between the patient and control groups in terms of their Treg cell values. When the epileptic patients were compared in terms of seizure type, there were also no differences in terms of the Treg cell values in patients with focal or generalized seizures.

## 6. Conclusions

This study is one of the limited studies in the literature to examine the effects of LEV monotherapy on the CBC and on humoral and cellular immunity, and is the first study to examine LEV’s effects on Treg cells in epileptic children and adolescents. The present study demonstrated that LEV monotherapy did not increase the incidence of infection, and there were no significant effects on the humoral or cellular immune system in epileptic children. These findings also suggest that the CD4/CD8 ratio among the lymphocyte subgroups was lower in patients with focal seizures. However, this study was limited due to its relatively small sample size. We believe that the immunological effect of LEV monotherapy in children with epilepsy needs to be investigated further via larger-scale randomized controlled trials.

## Figures and Tables

**Table 1:** Complete blood count values according to age in children.

Age (Year)	Hemoglobin g/dL	Leukocyte × 10^3^ μL	Neutrophil × 10^3^ μL	Lymphocyte × 10^3^ μL	Thrombocyte × 10^3^ μL
4–6	11.5–12.5	5–14.5	1.5–8.5	1.5–7	172–450
6–8				1.5–6.5	172–450
8–10				15–5.2	172–450
6–10		4.5–13.5	1.5–8		172–450
6–12	11.5–13.5				172–450
10–16		4.5–13	1.8–8	1–4.8	172–450
12–16	12–14				

**Table 2:** Demographic characteristics of the patient and control groups.

Groups	Epilepsy (n = 31)	Control (n = 43)	*p*
Age	8.82 ± 3.92	8.96 ± 3.39	0.86
Gender (female/male)	11/20	20/23	0.23

**Table 3:** Comparison of types of infections experienced by the patient and control groups in the first month of the study and comparison of frequency of infection in the first year of the study.

	Patient Group		Control Group		
**Types of infection in the last month**	n	%	n	%	*p*
Upper respiratory tract infection	8	80	8	72.7	0.39
Lower respiratory tract infection	1	10	1	9.1	
Urinary tract infection	0	0	2	18.2	
Acute gastroenteritis	1	10	0	0	
**Frequency of infection in the last one year**					
Had no infection	10	32.3	15	34.9	0.82
Had an infection once	13	41.9	15	34.9	
Had more than two infections	8	25.8	13	30.2	

**Table 4:** Relationship between the duration of LEV use, the doses at the beginning and at the end of treatment, and the frequency of infections in the last year.

Frequency of İnfection	Never	Once	Twice or More	*p*
Levetiracetam usage duration/year Median(minimum-maximum)	2 (1–4)	1 (1–2.5)	1.75 (1–4)	0.26
Treatment starting dose mg/kg/day Median(minimum-maximum)	20 (15–30)	20 (10–35)	25.5 (10–40)	0.52
Last dose used Median (minimum-maximum)	20 (13–36)	25 (15–40)	21 (12–29)	0.24

**Table 5:** Evaluation of complete blood count parameters in patient and control groups.

		Groups		
Blood Count Parameters		Patient n (%)	Control n (%)	*p*
Hemoglobin	Normal Low High	29 (%93.5) 2 (%6.5) 0 (%0)	35 (%81.4) 7 (%16.3) 1 (%2.3)	0.29
Leukocyte	Normal Low High	31 (%100) 0 (%0) 0 (%0)	41 (%95.3) 1 (%2.3) 1 (%2.3)	0.47
Lymphocyte	Normal Low High	28 (%90.3) 3 (%9.7) 0 (%0)	42 (%97.7) 1 (%2.3) 0 (%0)	0.30
Neutrophil	Normal Low High	29 (%93.5) 2 (%6.5) 0 (%0)	40 (%93) 3 (%7) 0	1.00
Platelet	Normal Low High	27 (%87.1) 3 (%9.7) 1 (%3.2)	41 (%95.3) 1 (%2.3) 1 (%2.3)	0.37

**Table 6:** Evaluation of immunoglobulin levels in patients with genetic, structural/metabolic and unknown cause epilepsy and in patients with focal seizure and generalized seizure.

Groups					
Immunoglobulin (Ig)		Genetic n (%)	Structural/Metabolic n (%)	Unknown Cause n (%)	*p*
Ig A level	Normal Low High	14 (%87.5) 0 (%0) 2 (%12.5)	9 (%100) 0 (%0) 0 (%0)	5 (%83.3) 0 (%0) 1 (%16.7)	0.48
Ig M level	Normal Low High	16 (%100) 0 (%0) 0 (%0)	8 (%88.9) 0 (%0) 1 (%11.1)	5 (%83.3) 0 (%0) 1 (%16.7)	0.29
Ig G level	Normal Low High	15 (%93.8) 0 (%0) 0 (%0)	9 (%100) 0 (%0) 0 (%0)	5 (%83.3) 0 (%0) 1 (%16.7)	0.26
Ig E level	Normal Low High	9(%56.2) 0 (%0) 7 (%43.8)	7 (%77.8) 0 (%0) 2 (%22.2)	2 (%33.3) 0 (%0) 4 (%66.7)	0.22
**Groups**					
**Immunoglobulin** **(Ig)**			**Focal seizure** **n (%)**	**Generalized seizure** **n (%)**	
Ig A level	Normal Low High		7 (%87.5) 0 (%0) 1 (%12.5)	21 (%91.3) 0 (%0) 2 (%8.7)	1.00
Ig M level	Normal Low High		8 (%100) 0 (%0) 0 (%0)	21 (%91.3) 0 (%0) 2 (%8.7)	1.00
Ig G level	Normal Low High		7 (%87.5) 0 (%0) 1 (%12.5)	22 (%95.7) 1 (%4.3) 0 (%0)	0.19
Ig E level	Normal Low High		5 (%62.5) 0 (%0) 3 (%37.5)	13 (%56.5) 0 (%0) 10 (%43.5)	1.00

**Table 7:** Evaluation of lymphocyte subsets in patient and control groups.

Groups				
		Patient n (%)	Control n (%)	*p*
CD_3_^+^	Normal Low High	27 (%87.1) 0 (%0) 4 (%12.9)	35 (%81.4) 0 (%0) 8 (%18.6)	0.75
CD_4_^+^	Normal Low High	28 (%90.3) 2 (%6.5) 1 (%3.2)	26 (%83.7) 4 (%9.3) 3 (%7)	0.69
CD_8_^+^	Normal Low High	28 (%90.3) 0 (%0) 3 (%9.7)	35 (%81.4) 2 (%4.7) 6 (%14)	0.39
CD_4_/CD_8_ ratio	Normal Low High	24 (%77.4) 7 (%22.6) 0 (%0)	35 (%81.4) 7 (%16.3) 1 (%2.3)	0.56
CD_19_^+^	Normal Low High	26 (%83.9) 5 (%16.1) 0 (%0)	36 (%83.7) 7 (%16.3) 0 (%0)	1.00
CD_56_^+^	Normal Low High	31 (%100) 0 (%0) 0 (%0)	39 (%90.7) 2 (%4.7) 2 (%4.7)	0.21
NKT	Normal Low High	30 (%96.8) 1 (%3.2) 0 (%0)	41 (%95.3) 2 (%4.7) 0 (%0)	1.00

**Table 8:** Evaluation of lymphocyte subset in patient focal and generalized seizure subgroups.

Groups				
		Focal seizure n (%)	Generalized Seizure n (%)	*p*
CD_3_^+^	Normal Low High	8 (%100) 0 (%0) 0 (%0)	19 (%82.6) 0 (%0) 4 (%17.4)	0.55
CD_4_^+^	Normal Low High	6 (%75) 2 (%25) 0 (%0)	22 (%95.7) 0 (%0) 1 (%4.3)	0.41
CD_8_^+^	Normal Low High	6 (%75) 0 (%0) 2 (%25)	22 (%95.7) 0 (%0) 1 (%4.3)	0.15
CD_4_/CD_8_ ratio	Normal Low High	3 (%37.5) 5 (%62.5) 0 (%0)	21 (%91.3) 2 (%8.7) 0 (%0)	0.006
CD_19_^+^	Normal Low High	7 (%87.5) 1 (%12.5) 0 (%0)	19 (%82.6) 4 (%17.4) 0 (%0)	1.00
CD_56_^+^	Normal Low High	8 (%100) 0 (%0) 0 (%0)	23 (%100) 0 (%0) 0 (%0)	–
NKT	Normal Low High	8 (%100) 0 (%0) 0 (%0)	22 (%95.7) 1 (%4.3) 0 (%0)	1.00

**Table 9:** Evaluation of CD4CD25foxP3% ratio and cell count per microliter in patient and control groups.

	Patient	Control	
	n = 31 Mean ± SD	n = 43 Mean ± SD	*p*
CD4CD25foxP3 (%)	0.07 ± 0.05	0.08 ± 0.06	0.78
CD4CD25foxP3 (μL)	5.37 ± 0.6	5.81 ± 0.8	0.70

## Data Availability

Data is contained within the article.

## Abbreviations

CNS: central nervous system; AED, antiepileptic drugs; URTI, upper respiratory tract infection; LEV, levetiracetam; CBC, complete blood count; Ig, immunoglobulin; VPA, valproic acid; KBZ, carbamazepine; Treg, Regulatory T Cell; NKT, Natural killer T cells; hb, hemoglobin.
